# PARylation at the Genome–Proteome Interface: Regulation of DNA Repair Protein Dynamics

**DOI:** 10.3390/ijms27198495

**Published:** 2026-09-23

**Authors:** Su-Jin Kang, Jiwon Eom, Yubin Ko, Soobin Yang, Sunbok Jang

**Affiliations:** 1 College of Pharmacy, Dongduk Women’s University, Seoul 02748, Republic of Korea; sjkang@dongduk.ac.kr; 2 College of Pharmacy, Graduate School of Pharmaceutical Sciences, Ewha Womans University, Seoul 03760, Republic of Korea; wonny529@ewha.ac.kr (J.E.); koyubin@ewha.ac.kr (Y.K.); binaida@ewha.ac.kr (S.Y.); 3 Graduate Program in Innovative Biomaterial Convergence, Ewha Womans University, Seoul 03760, Republic of Korea; 4 Graduate Program in Regulatory Science Convergence, Ewha Womans University, Seoul 03760, Republic of Korea

**Keywords:** PARylation, PARP1, nuclear proteostasis, DNA repair protein dynamics, biomolecular condensation, PARP inhibitors

## Abstract

Poly(ADP-ribosyl)ation (PARylation) is a rapid, reversible DNA damage response that promotes chromatin remodeling and the recruitment of repair factors. Genome maintenance also involves regulating the assembly, chromatin residence, exchange, and disengagement of repair proteins after lesion processing. This review examines PARylation through the lens of repair protein dynamics, distinguishing direct poly(ADP-ribose) (PAR)-dependent regulation from PAR-associated events and broader nuclear protein quality-control pathways. The strongest evidence concerns recruitment and chromatin residence. PAR binding drives XRCC1 accumulation at base excision repair (BER) and single-strand break (SSB) intermediates. In contrast, auto-PARylation promotes PARP1 release, and PARP inhibition favors persistent PARP1–DNA complexes that may require SUMO–RNF4–UFD1–p97/VCP-dependent extraction. In nucleotide excision repair (NER), PARP1-associated DDB2 regulation, ALC1 recruitment, and chromatin remodeling intersect with ubiquitin-dependent DDB2–XPC exchange. PAR-dependent condensation may add a spatial layer of protein organization, although its contribution remains context-dependent. We further discuss implications for PARP- and PARG-directed therapy and research-stage functional readouts. Together, these observations define a testable proteostatic-rheostat framework in which variable PAR signals influence the recruitment of selected repair proteins and subsequent chromatin residence; meanwhile, later extraction and turnover involve distinct, intersecting quality-control mechanisms.

## 1. Introduction

Maintaining genome integrity is essential for cellular homeostasis and organismal survival. Thus, to counteract continuous exposure to endogenous and exogenous genotoxic stress, cells rely on coordinated DNA damage response (DDR) pathways that detect DNA lesions, remodel chromatin, recruit repair factors, and restore genomic integrity. Defects in these processes promote genomic instability, a hallmark of cancer that contributes to tumor initiation, progression, and therapeutic resistance [1,2]. Although the enzymatic reactions of DNA repair are well characterized, successful repair also depends on regulating the behavior of repair proteins after each protein reaches the damaged chromatin. Notably, repair factors must assemble at appropriate sites, remain long enough to function, exchange with downstream components, and disengage after processing the lesion. Meanwhile, failure to regulate these events can leave persistent protein–DNA complexes that obstruct replication, transcription, or chromatin restoration [3,4].

Several types of DNA discontinuities rapidly activate poly(ADP-ribose) polymerase 1 (PARP1), which uses nicotinamide adenine dinucleotide (NAD^+^) to modify PARP1 and nearby proteins with poly(ADP-ribose) (PAR) [3,4,5,6]. This reversible modification, termed poly(ADP-ribosyl)ation (PARylation), promotes chromatin remodeling and the recruitment of repair factors [3,5,7]. Auto-PARylation also reduces the affinity of PARP1 for DNA, whereas PAR glycohydrolase (PARG)-mediated PAR hydrolysis terminates many PAR-dependent interactions. Consequently, PAR metabolism can influence both the formation and persistence of repair-associated protein assemblies [4,6,8,9]. These functions contribute to base excision repair (BER), single-strand break (SSB) repair, nucleotide excision repair (NER), double-strand break (DSB) responses, and the processing of replication-associated DNA damage [3,4,6,10].

Previous reviews have extensively addressed PARylation as a signal for DNA damage, a regulator of chromatin remodeling and the recruitment of repair factors, and a determinant of responses to PARP inhibitors (PARPi) [3,5,6,10]. Thus, this review examines the behavior of repair proteins after initial recruitment, focusing on changes in chromatin residence, molecular interactions, disengagement, extraction, recycling, and abundance. By organizing these processes around the changing states of individual repair proteins, this review provides a complementary perspective on how PAR signaling is integrated with chromatin remodeling and protein quality-control mechanisms during repair. These processes are related but do not follow a uniform or linear pathway. Proteins released after repair may return to the soluble nuclear pool, whereas persistent chromatin-bound complexes may require ATP-dependent extraction or proteolytic removal [11,12].

This perspective situates the dynamics of repair proteins within nuclear proteostasis, defined in this review as the mechanisms that maintain the functional availability, conformational competence, solubility, appropriate abundance, and chromatin association of nuclear proteins. PAR directly controls some of these properties through covalent ADP-ribosylation or non-covalent recognition by PAR-binding proteins. Chaperones, ubiquitin ligases, ATP-dependent segregases, and the proteasome regulate additional aspects of quality-control mechanisms for repair proteins [11,12,13]. The resulting framework distinguishes direct PAR-dependent regulation from PAR-associated processes and broader quality-control mechanisms that converge on the same repair proteins. This distinction allows recruitment, residence, extraction, and turnover to be compared without assuming that each protein belongs to a single PAR-initiated pathway.

BER and NER provide complementary systems for examining this distinction. In BER and SSB repair, PAR directly recruits X-ray repair cross-complementing protein 1 (XRCC1) and helps regulate PARP1 residence at repair intermediates [14,15,16,17,18]. In NER, PARP1 acts more indirectly through DNA damage-binding protein 2 (DDB2)-associated chromatin remodeling and the recruitment of the chromatin remodeler amplified in liver cancer 1 (ALC1). Subsequent exchange from DDB2 to xeroderma pigmentosum group C-complementing protein (XPC) depends largely on ubiquitin-mediated regulation [19,20,21,22]. These pathways illustrate varying degrees of direct PAR dependence while sharing the requirement for regulated protein recruitment, residence, remodeling, and removal. DSB repair and replication-associated damage provide additional contexts in which PARP1 influences competition among repair factors, DNA-end processing, and replication fork responses [23,24,25,26,27].

This review employs the term “proteostatic rheostat” as a conceptual framework to assess whether variables such as PAR abundance, polymer architecture, engagement of PAR-binding proteins, and signal duration produce graded changes in the recruitment or chromatin residence of selected repair proteins [8,28]. Rather than proposing a universal pathway for protein fate, this framework provides a basis for comparing how quantitatively variable PAR signals influence defined protein states and how these states intersect with distinct quality-control systems. The strongest experimental support for this framework is currently derived from studies of recruitment and chromatin residence, whereas the links between PAR properties and subsequent processes such as extraction, recycling, and degradation remain less clearly defined [11,12,14,16,29].

Since this is a narrative review, the literature search focused on primary mechanistic studies addressing PAR metabolism, the dynamics of DNA repair proteins, nuclear protein quality control, biomolecular condensation, and PARP-directed cancer therapy [3,5,6]. PubMed and Google Scholar were searched through 16 September 2026, and recent reviews and the reference lists of relevant primary studies were used to identify additional publications. Primary studies providing direct biochemical, cellular, or functional evidence were prioritized when evaluating mechanistic relationships. For consistency across the review, direct PAR-dependent regulation refers to protein-specific effects experimentally linked to PAR binding, ADP-ribosylation, or selective perturbation of PAR synthesis or hydrolysis [5,6]. Effects occurring within a PAR-regulated repair environment without a defined molecular connection to the protein are described as PAR-associated, whereas chaperone-, ubiquitin-, extraction-, or degradation-dependent processes without demonstrated PAR initiation are considered broader proteostatic mechanisms [11,12,13]. Figure 1 summarizes this framework by separating transient PAR signal generation and removal from experimentally defined changes in repair-protein recruitment and chromatin residence, and by showing how these PAR-dependent events intersect with broader nuclear protein quality-control pathways [8,9,11,12].

## 2. Molecular Determinants of PAR-Dependent Repair-Protein Dynamics

### 2.1. PAR Synthesis, Architecture, and Multivalent Interactions

PARylation can alter protein behavior because of the chemical and structural properties of PAR. Unlike many post-translational modifications (PTMs) that add a single chemical group to a defined residue, PARP1 and poly(ADP-ribose) polymerase 2 (PARP2) can initiate ADP-ribosylation on acceptor proteins and extend the modification into linear or branched polymers. Successive ADP-ribose units are linked by ribose–ribose glycosidic bonds, and branching creates additional sites for polymer extension. Variation in substrate identity, acceptor residue, chain length, and branching creates structurally heterogeneous PAR signals that can differ in their interactions with surrounding proteins [28]. During the DDR, PARP1 and PARP2 self-modify, as well as modify histones and other chromatin-associated proteins [7,30]. Histone PARylation factor 1 (HPF1) directs PARP1/2 activity toward serine residues and alters catalytic output by favoring initiation while restricting extensive chain elongation [30,31]. Thus, PAR abundance and architecture at damaged chromatin reflect the combined effects of substrate availability, PARP activation, NAD^+^ availability, HPF1, and PAR hydrolases. Some ADP-ribose-binding proteins distinguish mono-ADP-ribose from PAR or show preferences for polymer length or branching. How these structural differences affect the subsequent behavior of individual BER or NER proteins remains less well defined than their effects on PAR recognition [8,9,21].

The high negative charge density of PAR enables electrostatic interactions with positively charged protein surfaces and nucleic acid-binding regions. PAR also engages specialized recognition modules, including macrodomains, tryptophan–tryptophan–glutamate (WWE) domains, PAR-binding zinc fingers, and PAR-binding motifs [5,8,32]. Covalent ADP-ribosylation and non-covalent PAR binding can regulate different proteins within the same damage response: a factor may be modified, bind directly to PAR, or respond indirectly to PAR-dependent changes in chromatin structure and local protein interactions. PAR can also compete with DNA or RNA for selected nucleic acid-binding proteins, thereby altering their distribution between nucleic acids and PAR-rich regions [33,34,35]. These properties provide molecular routes through which PAR changes local protein concentrations and protein–protein or protein–nucleic acid interactions. Their consequences remain protein- and context-dependent rather than constituting a common downstream fate pathway. The contribution of multivalent PAR-dependent interactions to biomolecular condensation is discussed separately in Section 4 [33,34,35,36].

### 2.2. PAR Recognition and Signal Termination

PAR-dependent protein interactions arise from damage-induced polymer synthesis, recognition of ADP-ribose structures, and enzymatic removal of signals [8,9]. PARP1 is the predominant DNA damage-activated PAR polymerase, whereas PARP2 has overlapping but non-identical functions. DNA binding induces conformational rearrangements that activate PARP1, triggering auto-PARylation and PARylation of nearby proteins. PARP2 preferentially responds to DNA structures with phosphorylated termini and contributes to ADP-ribosylation at repair intermediates [3,4,5,30]. Auto-PARylation reduces PARP1 affinity for damaged DNA and facilitates PARP1 dissociation [29,37]. This reaction directly couples PAR synthesis to PARP1 residence time. PARPi suppress catalytic activity and can prolong PARP1–DNA association, although trapping also depends on inhibitor structure, PARP1 allostery, DNA substrate, and cellular context [29,38,39,40]. Meanwhile, histone and other chromatin-associated protein modifications create binding sites for PAR-responsive factors and remodelers. Thus, PARP activation can promote recruitment of one set of proteins while facilitating the release of PARP1 from the lesion [3,7,29,30].

PAR-binding modules recognize distinct structural features of ADP-ribose. WWE domains recognize internal iso-ADP-ribose linkages, whereas macrodomains, zinc-fingers, and motif-based interactions use distinct combinations of electrostatic and structural recognition [8,28,32]. These differences provide a molecular basis for protein-specific responses to PAR; however, quantitative relationships among polymer architecture, binding affinity, and residence at endogenous DNA lesions remain unavailable for most repair factors [28]. PARG hydrolyzes ribose–ribose bonds within PAR and accounts for most PAR-chain degradation, whereas ADP-ribosylhydrolase 3 (ARH3) efficiently removes serine-linked terminal ADP-ribose from proteins [8,9,41]. The complementary activities of these enzymes regulate different chemical components of signal persistence. PAR accumulation after DNA damage is generally rapid and transient, but its kinetics vary with lesion type, damage dose, cell type, and assay conditions. For this reason, protein-specific recruitment and residence measurements are more informative than a single generalized rate of PAR synthesis or degradation [4,9,29].

### 2.3. PAR-Dependent Recruitment and Chromatin Residence

XRCC1 and PARP1 serve as prominent examples of direct PAR-dependent regulation of repair-protein dynamics. XRCC1 functions as a non-catalytic scaffold within the BER and SSB repair pathways, with its first BRCA1 C-terminal (BRCT1) domain binding to PAR and enabling rapid accumulation at SSB intermediates. The inhibition of PAR synthesis or the disruption of PAR binding reduces XRCC1 recruitment. PAR recognition therefore directly determines the initial localization of XRCC1 rather than acting solely through PARP1-dependent chromatin remodeling [14,15,16,17]. XRCC1 then organizes DNA polymerase β (Pol β), DNA ligase III, and several end-processing enzymes [15,16,42]. The pathway-specific consequences of these interactions are addressed in Section 5. PARP1 residence is regulated by both its catalytic state and the persistence of the underlying DNA intermediate. Auto-PARylation promotes dissociation, whereas catalytic inhibition can stabilize PARP1–DNA complexes [29,37,38,39,40]. XRCC1-associated repair also removes DNA structures that sustain PARP1 binding. PARP1 residence reflects the combined effects of auto-modification and the rate at which downstream BER enzymes process the lesion [16,17,18].

The strongest mechanistic basis for the proteostatic-rheostat model currently lies in PAR-dependent recruitment and chromatin residence. PAR hydrolysis can terminate non-covalent interactions, thereby facilitating dissociation. Conversely, persistent protein–DNA complexes may require ATP-dependent extraction or proteolytic processing [8,9,11,12]. Nonetheless, whether PAR architecture and signal duration quantitatively influence these later outcomes remains an open mechanistic question [8,9,12,28].

## 3. Interfaces Between PAR Signaling and Nuclear Protein Quality Control

### 3.1. Chaperone Control of DNA Repair Proteins

Molecular chaperones maintain the folding, stability, and functional interactions of numerous genome-maintenance proteins. Heat shock protein 90 (HSP90) contributes to the stability or activity of breast cancer gene 1 (BRCA1), breast cancer gene 2 (BRCA2), RAD51, ataxia telangiectasia and Rad3-related (ATR), checkpoint kinase 1 (CHK1), and DNA-dependent protein kinase catalytic subunit (DNA-PKcs), thereby influencing homologous recombination (HR), checkpoint signaling, and DNA-end repair [13]. BER provides a more focused example of how chaperone control intersects with PAR-dependent repair mechanisms. HSP90 stabilizes XRCC1 and supports XRCC1–Pol β complex integrity, while the recruitment of XRCC1 to SSB intermediates depends directly on PAR [14,15,16,42]. These pathways regulate distinct aspects of the same repair process. PAR governs lesion-site recruitment of XRCC1, while HSP90 contributes to maintaining XRCC1 stability and XRCC1–Pol β complex integrity [14,15,16,42]. Current evidence does not place HSP90 downstream of PAR through a defined PAR-binding or PAR-dependent recruitment mechanism. Instead, PAR-dependent localization and chaperone-dependent complex maintenance represent intersecting mechanisms of repair-protein regulation.

### 3.2. PAR-Dependent Ubiquitination and Repair-Protein Turnover

PAR-dependent ubiquitination (PARdU) provides a direct biochemical link between ADP-ribosylation and the ubiquitin system [11,32]. RING finger protein 146 (RNF146) contains a WWE domain that recognizes internal iso-ADP-ribose linkages within PAR [32]. PAR binding relieves autoinhibition of its RING E3 ligase domain and enables ubiquitination of PARylated or PAR-associated substrates [43]. The best-characterized substrate-level examples arise from tankyrase signaling. Tankyrase-dependent PARylation of axis inhibition protein (AXIN) and SH3 domain-binding protein 2 (3BP2) promotes RNF146 recognition, leading to ubiquitination and subsequent degradation [44,45]. These processes establish a direct molecular pathway through which PAR functions as a recognition signal for ubiquitin-dependent protein processing.

RNF146-mediated PARdU can also target selected proteins involved in the DNA damage response. Biochemical and cellular studies have shown PARdU of PARP1, XRCC1, DNA ligase III, and Ku70, indicating that this mechanism is not restricted to tankyrase-regulated signaling pathways [46]. However, the extent to which RNF146 determines the endogenous chromatin residence, extraction, or turnover of these proteins during BER, SSB repair, or other repair processes remains inadequately characterized [11,46]. Therefore, PARdU should be distinguished from the broader set of ubiquitin-dependent mechanisms that regulate repair proteins independently of a demonstrated RNF146–PAR recognition step.

This distinction is particularly important for Pol β, flap endonuclease 1 (FEN1), mutY DNA glycosylase (MUTYH), xeroderma pigmentosum group A-complementing protein (XPA), and DDB2, whose ubiquitin-dependent regulation is well documented but is mediated through other E3 ligase pathways or has not been directly linked to RNF146 recognition. Pol β abundance is regulated by C terminus of Hsc70-interacting protein (CHIP) and Mule/alternative reading frame-binding protein 1 (ARF-BP1); FEN1 turnover is governed by sequential PTMs; MUTYH stability is controlled by Mule; XPA localization and stability are regulated by ATR-dependent mechanisms and by ubiquitination mediated by the HECT and RLD domain-containing E3 ubiquitin protein ligase 2 (HERC2); DDB2–XPC exchange and DDB2 turnover are mediated by NER-associated ubiquitin pathways [19,22,47,48,49,50,51]. These ubiquitin- and PTM-dependent mechanisms can alter protein stability, chromatin association, or factor exchange without requiring PAR to serve as the initiating signal. Thus, PARdU represents one specific route connecting PAR recognition to ubiquitination. In contrast, the turnover of repair proteins more broadly reflects the combined actions of multiple ubiquitin-dependent quality-control pathways with different relationships to PAR signaling [11].

### 3.3. p97/VCP-Dependent Extraction of Trapped PARP1

Persistent protein–DNA complexes may require active removal mechanisms that differ from spontaneous dissociation or regulation of steady-state protein abundance. PARP1 provides a clear example. Auto-PARylation normally reduces the affinity of PARP1 for damaged DNA, whereas catalytic inhibition can stabilize PARP1–DNA complexes and create physical obstacles to replication and transcription [38,39,40]. Trapped PARP1 can be processed through a small ubiquitin-like modifier (SUMO)- and ubiquitin-dependent extraction pathway. SUMOylated PARP1 is recognized by RING finger protein 4 (RNF4), which recruits the AAA ATPase p97/valosin-containing protein (p97/VCP) via ubiquitin fusion degradation protein 1 (UFD1), enabling extraction of PARP1 from chromatin. Impairment of this pathway prolongs PARP1 retention and increases sensitivity to PARP inhibition [12]. This extraction pathway is mechanistically distinct from RNF146-mediated PARdU. RNF146 recognizes PAR and couples PAR recognition to ubiquitination, whereas RNF4 responds to SUMO-dependent signals associated with persistently bound PARP1 after catalytic inhibition [11,32,43,46]. Since extraction does not necessarily cause immediate degradation, p97/VCP-dependent removal should be distinguished from proteolytic turnover.

### 3.4. Stress-Responsive PAR Signaling and Protein Organization

PAR signaling also alters protein–nucleic acid interactions during cellular stress, extending its influence beyond canonical lesion processing. During heat shock, PARP1-dependent modification of poly(A) polymerase reduces its RNA-binding capacity and suppresses polyadenylation of non-heat-shock transcripts [52]. Furthermore, PAR can modulate the recruitment and mobility of stress-responsive RNA-binding proteins, including fused in sarcoma (FUS), TAR DNA-binding protein 43 (TDP-43), and heterogeneous nuclear ribonucleoprotein A1 (hnRNP A1), in reconstituted systems and cellular stress granules [35,53,54]. These examples demonstrate that ADP-ribosylation can rapidly alter protein interaction states and local organization in response to stress [52,55,56].

These stress-responsive systems are informative as comparative models rather than direct surrogates for DNA-repair assemblies. Proteotoxic or transcriptional stress can engage PARP1 through secondary oxidative DNA damage, transcription-associated lesions, or replication stress, and the resulting changes in protein organization remain strongly context-dependent [3,4,55]. Together with chaperone control, ubiquitin-dependent regulation, and p97/VCP-mediated extraction, these observations illustrate distinct points at which PAR signaling intersects with nuclear protein quality control without requiring a single PAR-initiated pathway [11,12,13]. Against this background, Section 4 considers when multivalent PAR–protein interactions generate molecular assemblies that satisfy biophysical criteria for biomolecular condensation.

## 4. PAR-Dependent Biomolecular Condensation in the DDR

### 4.1. Physical Basis of PAR-Dependent Condensation

PAR creates a highly charged, multivalent molecular environment that can concentrate proteins and nucleic acids through electrostatic and domain-specific interactions. At DNA damage sites, these interactions can increase local molecular concentration and, under appropriate conditions, promote biomolecular condensation. The resulting assemblies are physically distinct from simple recruitment and exhibit collective properties, including concentration dependence, saturation, reversible molecular exchange, selective partitioning, and dense-phase formation [33,34,36,57]. Reconstituted systems are particularly informative for characterizing these properties because protein, DNA, PAR, and buffer concentrations can be controlled directly. Cellular assemblies are more complex because chromatin attachment, RNA, molecular crowding, and additional PTMs influence local protein behavior. Therefore, combining biochemical reconstitution with quantitative cellular measurements is necessary to relate PAR-dependent condensation to DNA repair under physiological conditions [36,57,58].

Fluorescence recovery after photobleaching (FRAP), single-particle tracking, and related imaging methods offer valuable insights into molecular mobility and exchange. These measurements complement structural and concentration-dependent criteria; however, they do not, in isolation, establish the physical state of an assembly. In DNA repair, the analysis of condensates is most informative for determining how PAR-dependent multivalency changes local protein concentration, composition, and exchange kinetics [33,34,36,58].

### 4.2. PARP1–DNA Co-Condensation and Protein Partitioning

Reconstitution experiments have shown that PARP1 and damaged DNA can form condensed assemblies under defined biochemical conditions. PARP1 binds DNA breaks through its N-terminal DNA-binding domains, and multivalent interactions among PARP1 and DNA molecules promote network formation [57]. Repair proteins partition into these assemblies to varying degrees, depending on properties such as DNA binding, PAR affinity, molecular valency, and interactions with other components. Thus, PARP1–DNA condensation can generate a locally enriched yet compositionally selective repair environment [36,57,58]. PAR synthesis further changes the composition of this environment. Auto-PARylated PARP1 has reduced affinity for damaged DNA, and newly synthesized PAR creates binding sites for PAR-responsive proteins. Consequently, PARP1 activation can redistribute interactions within the assembly by weakening PARP1–DNA contacts while increasing recruitment of PAR-binding factors [3,4,36,57].

XRCC1 illustrates how this process interfaces with a well-defined DNA repair mechanism. Its PAR-binding BRCT1 domain drives rapid accumulation at SSB intermediates, linking local PAR synthesis directly to XRCC1 concentration at damaged chromatin [14,15,16]. Whether this enrichment also involves partitioning into a distinct condensed phase is a separate physical question; however, the established interaction between PAR and XRCC1 provides a mechanistic route through which PAR-rich assemblies can alter repair-protein composition [36,57,58].

### 4.3. Molecular Exchange and PAR-Dependent Assembly Dynamics

The functional behavior of a repair-associated assembly depends not only on its composition but also on the rates at which individual proteins enter, remain, and leave. FRAP can estimate mobile fractions and recovery kinetics, while single-particle tracking and fluorescence correlation approaches provide complementary measurements of diffusion and residence durations. These parameters depend on factors such as protein identity, lesion density, expression levels, and the local chromatin environment, making protein-specific kinetic measurements more informative than a single generalized timescale for PAR-dependent assemblies [29,36,57,58].

PAR synthesis and hydrolysis provide a reversible mechanism for changing these interactions. PARP1 activation rapidly increases the local number of PAR-binding sites, whereas PARG-mediated hydrolysis progressively removes them [8,9]. This cycle can shift both the concentration and residence of PAR-responsive proteins as repair proceeds. Auto-PARylation simultaneously decreases PARP1–DNA affinity, so PAR synthesis can promote recruitment of PAR-binding proteins while facilitating PARP1 dissociation [4,29,39,40]. This combination of opposing effects provides a mechanistic basis for dynamic changes in assembly composition. Rather than driving repair proteins through a common sequence, PAR metabolism modulates individual molecular interactions according to their dependence on DNA, PAR, and other components of the repair complex [36,57,58].

Liquid-to-gel or liquid-to-solid transitions have been most extensively characterized in PAR-responsive ribonucleoprotein systems, including proteins such as FUS and TDP-43 [35,53,54]. These systems demonstrate that PAR can influence molecular mobility and material properties over time. Comparable transitions remain less well characterized at BER and NER sites, where recruitment and chromatin residence currently provide the more firmly defined PAR-dependent processes [14,15,16,20,21,22].

### 4.4. Condensation in the Context of BER and NER

The contribution of biomolecular condensation to DNA repair is likely to vary among pathways and protein assemblies. In BER and SSB repair, PAR-dependent recruitment of XRCC1 provides a well-defined molecular mechanism for concentrating a repair scaffold at lesion intermediates, and PARG-mediated PAR hydrolysis correlates with subsequent reduction in XRCC1 enrichment [14,15,16]. These observations establish a dynamic, PAR-dependent recruitment process; however, these findings do not, in themselves, demonstrate that the complete XRCC1-centered repair complex forms a distinct condensed phase. Whether condensation provides additional control over XRCC1 residence, molecular exchange, or repair efficiency remains an independent biophysical question [36,57,58].

A similar distinction applies to NER. PARP1-dependent recruitment of ALC1 and the resulting chromatin remodeling facilitate early factor accumulation, whereas DDB2–XPC exchange is largely regulated through ubiquitin-dependent mechanisms [20,21,22]. Although these processes occur within the same lesion-associated chromatin environment, local enrichment and coordinated factor exchange are insufficient to establish condensation. Trapped PARP1 represents an even more distinct protein state. Prolonged PARP1–DNA association generates a persistent chromatin-bound complex that can require p97/VCP-dependent extraction, rather than a dynamic assembly defined by reversible molecular exchange [12,38,39,40].

PAR-dependent condensation should be viewed as an additional spatial and biophysical layer of repair-protein regulation, rather than a general mechanism underlying the recruitment or removal of repair factors. The functional significance of PAR-dependent condensation will be best established by combining defined biochemical reconstitution with quantitative measurements of endogenous protein concentration, partitioning, mobility, and residence in physiologically relevant repair settings [33,34,35,36,57]. Such analysis will clarify when condensation alters the behavior of repair proteins beyond the effects attributable to direct PAR binding, chromatin remodeling, or other protein-quality-control mechanisms [14,20,21,22].

## 5. Pathway-Specific Roles of PAR in Repair-Protein Dynamics

Figure 2 compares the dynamics of repair proteins in BER/SSB repair and NER, highlighting direct PAR-dependent XRCC1 recruitment and PARP1 residence in BER/SSB repair versus PARP1-associated chromatin remodeling and regulated DDB2–XPC exchange in NER. Table 1 complements this pathway-level comparison by summarizing selected repair proteins according to their relationship to PAR signaling, experimental basis, protein-dynamic consequence, and associated proteostatic mechanism.

### 5.1. XRCC1 Recruitment and PARP1 Residence in BER and SSB Repair

In BER and SSB repair processes, the processing of damaged bases generates SSB intermediates that can activate PARP1 and PARP2 [15,16,59]. The extent and duration of PARP activation depend on the structure and persistence of these intermediates, linking downstream BER capacity to local PAR synthesis and XRCC1 recruitment [15,16]. Notably, XRCC1 provides the strongest example of direct PAR-dependent recruitment in this pathway [14,15,16], with its BRCT1 domain binding to PAR and facilitating rapid accumulation at DNA breaks [14]. Subsequently, XRCC1 orchestrates the assembly of Pol β, DNA ligase III, polynucleotide kinase 3′-phosphatase (PNKP), aprataxin, and other end-processing factors [15,16]. PARG-mediated PAR hydrolysis progressively removes XRCC1-binding sites and contributes to declining XRCC1 enrichment after lesion processing [8,9,15]. PARP1 residence is coupled reciprocally to this process. PARP1 generates the PAR required for XRCC1 recruitment, whereas XRCC1-associated Pol β and DNA ligase III process and seal the same DNA intermediates, reducing the structures that sustain PARP1 binding. When SSB formation exceeds the available BER capacity, PARP1 can bind otherwise unprocessed SSBs, potentially protecting these intermediates from nuclease attack and limiting their conversion into DSBs [18]. However, in conditions where downstream BER processing remains impaired, persistent engagement of PARP1 can instead promote prolonged PARP1 activation and trapping [16,17,18].

Several BER enzymes are also regulated through mechanisms that primarily affect protein stability, chromatin association, or turnover rather than direct PAR-dependent recruitment. Pol β abundance is regulated by ubiquitin ligases including Mule/alternative reading frame-binding protein 1 (ARF-BP1) and CHIP, while FEN1 undergoes sequential phosphorylation, SUMOylation, and ubiquitination that influence chromatin association and turnover [47,48,49]. Mule-dependent ubiquitination also regulates MUTYH stability, whereas apurinic/apyrimidinic endonuclease 1 (APE1) and 8-oxoguanine DNA glycosylase 1 (OGG1) interact functionally with PARP1 during processing of BER intermediates [50,59,60]. These mechanisms provide additional control over BER capacity alongside the more direct PAR-dependent regulation of XRCC1 and PARP1 residence.

### 5.2. Chromatin Remodeling and DDB2–XPC Exchange in NER

NER removes bulky, helix-distorting lesions, including ultraviolet (UV)-induced cyclobutane pyrimidine dimers and 6-4 photoproducts. Global genome NER (GG-NER) begins with lesion detection by the UV-damaged DNA-binding (UV–DDB) complex and XPC, followed by verification by transcription factor IIH (TFIIH), dual incision, gap-filling DNA synthesis, and ligation. PARP1 primarily contributes during the early recognition and chromatin-remodeling stages of this pathway [19,20,21,22].

DDB2, as part of the DNA damage-binding protein 1 (DDB1)–DDB2 complex, recognizes UV-induced photolesions and promotes XPC recruitment [19,20,22]. DDB2 also functions within the DDB2-containing cullin 4-RING E3 ubiquitin ligase complex (CRL4^DDB2), which modifies DDB2 and XPC as the pathway progresses from initial lesion recognition to XPC-centered verification. TFIIH recruitment further promotes DDB2 dissociation, and ubiquitinated DDB2 can then be released or degraded [19,22]. PARP1 acts within this early chromatin environment through its interaction with DDB2 and the PAR-dependent recruitment of the chromatin remodeler ALC1. Meanwhile, ALC1-mediated nucleosome remodeling increases lesion accessibility and facilitates recruitment of XPC and other NER factors [20,21]. Early NER combines PAR-dependent regulation of chromatin accessibility with ubiquitin-dependent control of factor exchange, allowing lesion recognition to proceed without prolonged occupancy by the initiating proteins [19,20,21,22].

XPA and transcription-coupled NER (TC-NER) introduce additional regulatory layers. XPA is modified by phosphorylation and ubiquitination, two processes that influence its activity, localization, and stability [51]. In TC-NER, stalled RNA polymerase II (RNAPII) recruits remodeling and ubiquitination pathways mediated by the Cockayne syndrome group B protein (CSB) and the Cockayne syndrome group A protein (CSA) [61,62]. Recent evidence further indicates that PARP1-dependent PARylation of CSB stabilizes its association with lesion-stalled RNAPII and modulates CSB ATPase activity, providing a direct PAR-dependent component in early TC-NER [63]. These mechanisms illustrate how PAR signaling and other post-translational regulatory pathways jointly control factor residence and exchange during NER [22,61,63].

### 5.3. DSB Repair and Replication-Associated Damage

PARP1 also responds to DSBs, and PARP1 activity is influenced by DNA-end structure, chromatin context, cell-cycle phase, and competition with other DNA-end-binding proteins. PARP1 and Ku70–Ku80 exemplify competition at DSBs; imaging and biochemical studies show they compete for DNA-end occupancy and are associated with distinct end-joining pathways [23,24]. Ku occupancy favors classical non-homologous end joining (NHEJ), whereas reduced Ku-dependent end joining increases the relative contribution of PARP1-associated alternative end joining [24]. This competition illustrates how the relative occupancy of repair proteins at DNA ends can influence pathway utilization without implying that subsequent end-processing reactions are uniformly PAR-dependent. Furthermore, PARP1-dependent chromatin remodeling and repair-site organization can further alter the accessibility of broken DNA ends [3,57].

Replication-associated damage provides a complementary setting in which PARP1 residence has direct physical consequences. PARP1 also influences homologous recombination (HR)-associated responses through chromatin remodeling, fork-associated signaling, and processing of lesions that become DSBs during S phase [3,4,25,26,27]. PARP inhibition impedes the maturation of nascent DNA strands, while unrepaired BER intermediates in template DNA can trigger replication fork collapse and sensitivity to PARPi [25,26]. Catalytic inhibition and PARP1 trapping affect these processes differently. Reduced synthesis of PAR limits the recruitment of PAR-responsive factors, whereas trapped PARP1 forms a physical obstacle to fork progression that must be remodeled or extracted [12,38,39,40]. Replication across or near these complexes can slow forks, generate single-stranded DNA gaps, and cause DNA breaks, which activate HR, fork-protection pathways, or end joining [25,26,27,38]. Thus, in DSB and replication-associated repair, the best-supported protein-dynamic effects involve competition, occupancy, and the persistence of PARP1 at DNA ends or replication-associated lesions, whereas direct PAR-dependent regulation of downstream HR proteins is comparatively less well established.

### 5.4. Comparative Features Across Repair Pathways

Comparison of these pathways reveals both shared principles and substantial mechanistic differences. PAR binding directly recruits XRCC1 during BER and SSB repair, and auto-PARylation regulates PARP1 dissociation [14,15,16,29,37]. In NER, PARP1 mainly contributes to early chromatin remodeling and factor recruitment, whereas ubiquitin-dependent mechanisms control much of the subsequent DDB2–XPC exchange [20,21,22]. At DSBs and stalled forks, PARP1 influences DNA-end competition, chromatin accessibility, and replication-associated processing [23,25,26,27]. Dissociation, extraction, and degradation also represent distinct molecular outcomes. Hydrolysis of PAR can terminate a PAR-dependent binding interaction and permit diffusion away from a lesion [8,9]. Persistent protein–DNA complexes may instead require ATP-dependent extraction, whereas ubiquitination and proteasomal degradation regulate protein abundance over a longer period [12,47,48].

Across these pathways, the primary common function of PAR is to alter the local availability and chromatin residence of selected proteins [4,8,9]. Subsequent processes such as remodeling, extraction, and turnover engage pathway-specific combinations of chromatin remodelers, ubiquitin ligases, segregases, and proteolytic machinery [11,12,32,42]. The proteostatic-rheostat framework is most informative when these processes are treated as interconnected yet mechanistically distinct determinants of repair-protein behavior.

## 6. Therapeutic Implications of PAR-Associated Repair-Protein Dynamics

### 6.1. PARP Inhibition, Repair-Factor Recruitment, and PARP1 Trapping

PARPi are clinically established treatments for selected ovarian, breast, pancreatic, and prostate cancers, particularly those with *BRCA1/2* alterations or other forms of homologous recombination deficiency (HRD) [64,65,66]. Their activity reflects several interacting mechanisms, including catalytic inhibition of PARP1/2, reduced PAR-dependent recruitment, replication-associated DNA damage, and PARP1 trapping. The relative contributions of these mechanisms vary among inhibitors, genetic backgrounds, lesion types, and replication states [25,26,38,39]. Suppressing PAR synthesis reduces recruitment of PAR-binding proteins such as XRCC1 without necessarily changing their total cellular abundance [14,15,16]. Catalytic inhibition also prevents auto-PARylation-mediated PARP1 release, while compound-specific allosteric effects can further stabilize PARP1–DNA complexes [38,39,40]. Thus, catalytic inhibition and trapping produce distinguishable changes in repair-protein dynamics despite arising from the same pharmacological target. Figure 3 places these clinically established PARP-directed mechanisms alongside emerging and exploratory vulnerabilities and research-stage functional readouts, highlighting their distinct levels of translational maturity.

Trapped PARP1 obstructs fork progression, transcription, and access by repair enzymes [25,26,38,40]. Replication near these complexes generates fork slowing, single-stranded DNA gaps, and DNA breaks, which are particularly toxic when HR or fork-protection pathways are defective [25,26,27,38]. The SUMO–RNF4–UFD1–p97/VCP-dependent extraction process provides a mechanism for removing trapped PARP1 and correlates the capacity for chromatin–protein extraction with PARPi response [12].

Established mechanisms of resistance include *BRCA1/2* reversion, restoration of HR, reduced PARP1 trapping, replication-fork protection, and altered drug transport [64,65]. Chromatin-associated adaptations can add another layer to this resistance landscape. Increased dependence on the histone chaperone, nuclear autoantigenic sperm protein (NASP) in PARPi-resistant models indicates that maintaining histone supply and chromatin organization can support survival during sustained PARP inhibition [67].

### 6.2. PAR Turnover and Trapped-Protein Extraction

PARG inhibition perturbs PAR metabolism in the opposite direction of PARP inhibition by prolonging the lifetime of PAR. In susceptible tumor models, sustained PAR interferes with recovery from replication stress and creates a distinct therapeutic liability. Conversely, loss of PARG can preserve residual PARylation during PARP inhibition and reduce PARPi sensitivity in some genetic contexts [68]. The therapeutic consequence of altered PAR degradation depends strongly on the underlying replication and PAR-metabolic state of the tumor. PARG inhibitors have entered early clinical development, although predictive biomarkers, tumor selectivity, and the therapeutic window remain to be defined [69,70]. Their development is particularly relevant to tumors in which persistent PAR signaling or defective recovery from replication stress creates a measurable dependency [69,71,72].

A complementary therapeutic concept arises from the removal of persistent PARP1–DNA complexes. The p97/VCP-mediated extraction has a more direct relationship to PARPi treatment because impaired extraction prolongs the chromatin residence of trapped PARP1. This creates a potential route for increasing trapping-associated toxicity, although the broad cellular functions of p97/VCP impose a major selectivity constraint. Therapeutic development will require settings in which tumor dependence on chromatin–protein extraction can be separated from essential p97/VCP functions in normal cells [12].

### 6.3. Chaperone Replication Stress, Emerging Biophysical Vulnerabilities

HSP90 regulates the stability or activity of several proteins required for HR, checkpoint signaling, and DNA-end repair, including BRCA1, BRCA2, RAD51, ATR, CHK1, and DNA-PKcs [12]. In selected ovarian and breast cancer models, HSP90 inhibition reduces HR capacity and increases sensitivity to platinum agents or PARPi [73,74]. HSP90-mediated stabilization of mutant BRCA1 provides a defined setting in which chaperone dependence can maintain a repair-competent and drug-resistant state [75]. HSP90 inhibition acts across a broad protein network rather than selectively targeting PAR-associated repair. Therefore, combining HSP90 inhibition with PARPi is most compelling in tumors where chaperone-dependent stabilization of HR or checkpoint proteins directly contributes to resistance [13,73,74,75].

ATR and CHK1 inhibition target a related, more specific, replication-associated vulnerability. Indeed, by weakening checkpoint control, fork stabilization, or HR, these agents can exacerbate the consequences of replication stress induced by PARP inhibition. Such combinations illustrate how vulnerabilities arising from altered repair-protein states can be exploited through pathways outside PAR metabolism [27,76].

Therapeutic manipulation of repair-associated condensation occurs earlier. PARP and PARG inhibitors alter the abundance and lifetime of PAR, thereby changing PAR-containing assemblies; meanwhile, chaperone and transcriptional perturbations may influence protein partitioning through broader effects on the nuclear proteome. Establishing condensation as a druggable mechanism will require linking a defined physical change in a repair assembly to an independent effect on tumor response [33,34,36,57].

### 6.4. Dynamic Repair-Protein States as Research Biomarkers

The selection of patients for PARPi therapy currently relies mainly on BRCA1/2 mutations, HRD assays, genomic scars, prior sensitivity to platinum therapies, and the detection of reversion mutations during instances of acquired resistance [64,65,66,77,78]. These biomarkers describe genetic or historical features of repair deficiency but provide limited information about the immediate molecular state of PARP1 or other repair proteins during treatment. PAR abundance, PAR turnover, and PARP1 chromatin association provide complementary functional measurements [39,40]. PAR analysis requires rapid sample handling because synthesis and hydrolysis occur on short time scales. Furthermore, PARP1 trapping measurements depend on extraction conditions, lesion load, drug exposure, and assay format. The implementation of standardized sample processing and analytical procedures will be essential for accurately comparing these states across experimental and clinical specimens [64,65,66].

Recruitment kinetics of XRCC1 or DDB2 can be measured by live-cell imaging after controlled DNA damage, while FRAP and single-particle tracking provide measurements of mobility and residence [29,36,58]. Applying these methods to patient material is technically more demanding because these approaches generally require viable cells and controlled experimental perturbations. Patient-derived organoids and ex vivo cultures may provide useful intermediate systems for relating dynamic protein behavior to treatment response [12]. Chromatin proteomics, proximity labeling, ADP-ribosylation profiling, ubiquitinomics, and single-cell assays can define treatment-induced changes in repair-associated proteins on a broader scale [5,8]. Integration with genomic assays and functional measurements, such as RAD51 focus formation, may distinguish HR restoration from altered PARP1 trapping, protein extraction, or stabilization [77,78].

These measurements currently function primarily as research biomarkers. Their clinical value will depend on reproducibility, access to suitable tumor material, prospective association with therapeutic response, and incremental information beyond established *BRCA* and HRD testing [66,77,78]. A near-term application may be the mechanistic selection of combination therapies for tumors whose resistance cannot be adequately explained by genomic biomarkers alone [64,65,66]. Table 2 summarizes the translational spectrum of these approaches by comparing their mechanistic basis, current development status, relevant experimental or clinical settings, and principal limitations.

## 7. Conclusions and Future Directions

PARylation regulates several well-defined aspects of repair-protein behavior at damaged chromatin. PAR binding recruits XRCC1 during BER and SSB repair, whereas auto-PARylation reduces PARP1 residence on damaged DNA. Catalytic inhibition reverses this balance, favoring persistent PARP1–DNA complexes that can require SUMO-RNF4-UFD1-p97/VCP-dependent extraction. In NER, PARP1 contributes to ALC1 recruitment and chromatin remodeling associated with DDB2, whereas ubiquitin-dependent mechanisms control the subsequent exchange of lesion-recognition factors. These examples show that PAR signaling, chromatin remodeling, and nuclear protein quality control converge on repair-protein behavior through distinct molecular mechanisms.

On this basis, we propose the proteostatic-rheostat model as a testable conceptual framework rather than an established mechanism for graded regulation. A central, testable prediction of the model is that quantitative variation in PAR abundance, polymer architecture, or signal duration produces graded changes in the recruitment or chromatin residence of selected repair proteins. Current evidence is strongest for PAR-dependent recruitment and control of chromatin residence, whereas the relationships between quantitative PAR properties and later outcomes, such as extraction, recycling, and degradation, remain less well resolved. PAR-dependent condensation adds another level of spatial organization, although its contribution to repair-protein dynamics likely varies across repair pathways.

These predictions and unresolved relationships can now be tested quantitatively. Defined linear and branched PAR polymers can determine how polymer architecture affects the binding and residence of individual repair proteins. Endogenous tagging and live-cell imaging can measure how perturbations of PARP, PARG, or ARH3 alter recruitment and release kinetics; meanwhile, pulse-chase proteomics and chromatin fractionation can distinguish dissociation from p97/VCP-dependent extraction and proteolytic turnover. Reconstituted systems combined with physiologically relevant cellular models can determine when PAR-dependent multivalency produces a distinct condensed state and whether that state changes repair kinetics or protein exchange.

Extending these measurements to organoids and tumor models will clarify whether dynamic properties, such as PARP1 trapping, extraction capacity, or PAR turnover, provide information beyond established HRD biomarkers. These studies will determine which aspects of PAR-dependent regulation of repair proteins are broadly conserved and which remain pathway-specific. This distinction should sharpen mechanistic interpretation of PARP-directed therapy and may reveal therapeutic dependencies not captured by static DNA repair genotyping alone.

## Figures and Tables

**Figure 1:**
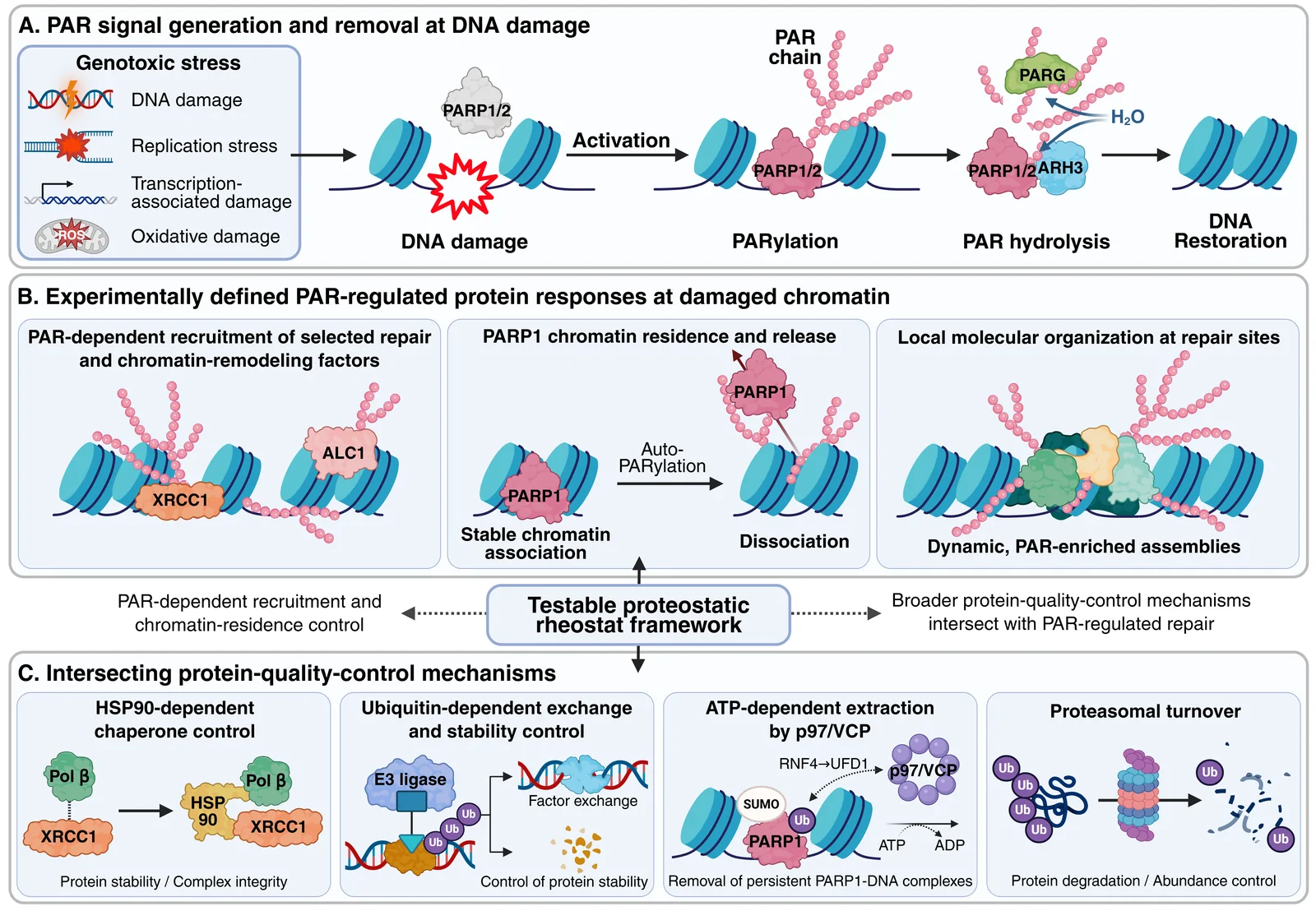
PAR-dependent regulation of DNA repair-protein dynamics at damaged chromatin. (**A**) Genotoxic stress activates PARP1/2, generating transient PAR signals that are reversed mainly by PARG and ARH3. (**B**) Experimentally defined PAR-regulated responses include recruitment of selected repair or chromatin-remodeling factors, such as XRCC1 and ALC1, auto-PARylation-dependent PARP1 release, and local PAR-enriched molecular organization. (**C**) These PAR-regulated events intersect with broader protein-quality-control mechanisms, including HSP90-dependent stabilization of repair proteins and complexes, ubiquitin-dependent control of protein exchange and stability, p97/VCP-dependent extraction of persistent chromatin-bound complexes, and proteasomal turnover. The proteostatic-rheostat concept is presented as a testable framework linking quantitatively variable PAR signaling to selected repair-protein states rather than as an established universal mechanism governing protein fate. Solid vertical arrows indicate integration of the experimentally defined and broader quality-control domains within the framework, whereas dotted horizontal arrows denote conceptual relationships rather than direct biochemical cusation. Abbreviations: ALC1, amplified in liver cancer 1; HSP90, heat shock protein 90; PARG, poly(ADP-ribose) glycohydrolase; RNF4, Ring finger protein 4; UFD1, ubiquitin fusion degradation protein 1, p97/VCP, p97/valosin-containing protein; XRCC1, X-ray repair cross-complementing protein 1. Created in Biorender. Jang, S. (2026) https://BioRender.com/9ozo2tu.

**Figure 2:**
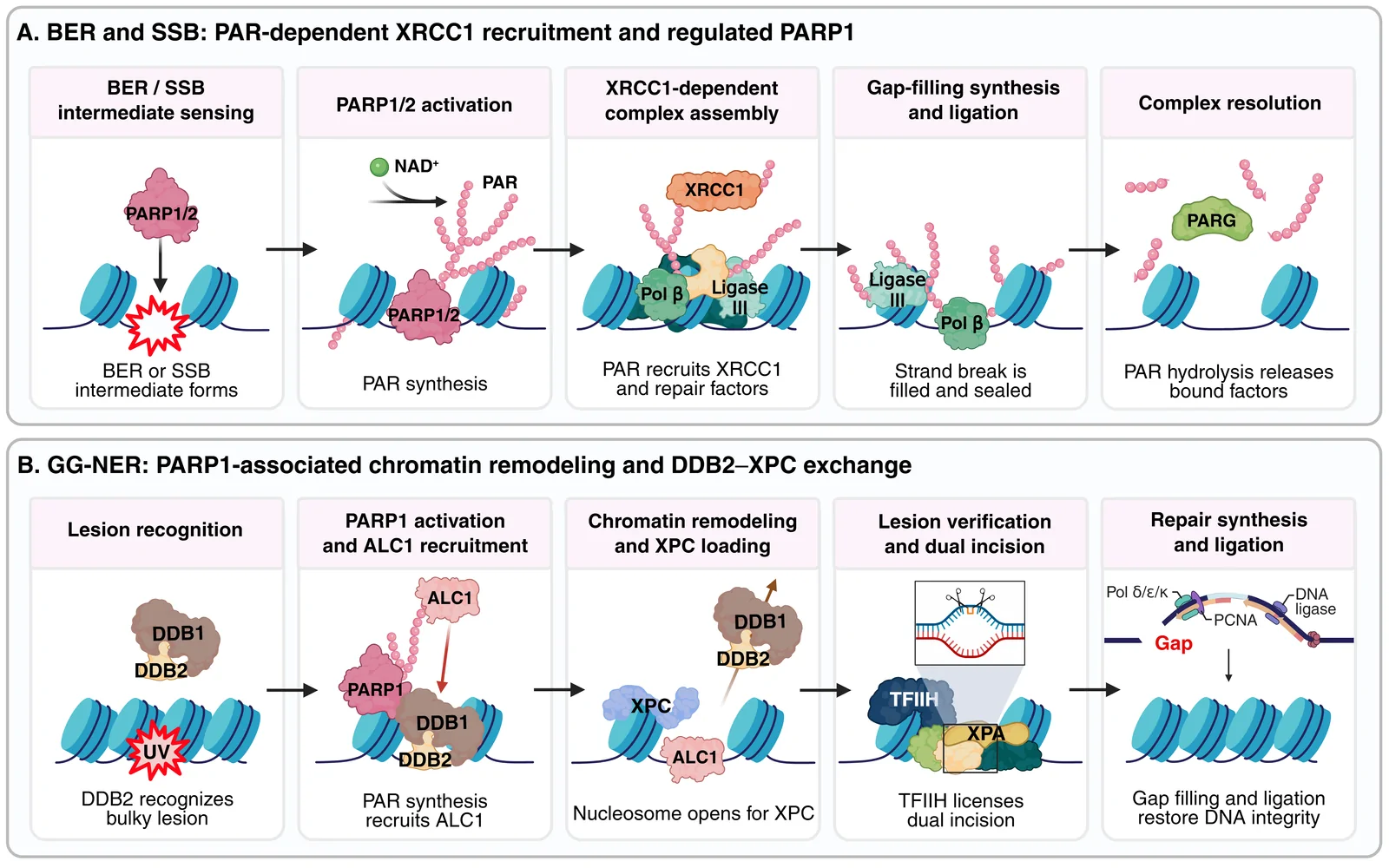
Representative pathway-specific roles of PAR in repair-protein dynamics during BER/SSB repair and GG-NER. (**A**) In BER and SSB repair pathways, PARP1/2-generated PAR promotes rapid recruitment of XRCC1-centered repair complexes containing Pol β, DNA ligase III, and other end-processing factors. Gap filling and ligation ligation DNA integrity, while PARG-mediated PAR hydrolysis promotes complex resolution and protein release. (**B**) In GG-NER, DDB2 recognizes bulky lesions, while PARP1-dependent PAR synthesis recruits ALC1 and promotes chromatin remodeling and XPC loading. TFIIH- and XPA-dependent lesion verification is followed by dual incision, repair synthesis across the resulting excision gap, and ligation to restore DNA integrity. Abbreviations: ALC1, amplified in liver cancer 1; BER, base excision repair; GG-NER, global genome nucleotide excision repair; DDB1, DNA damage-binding protein 1; DDB2, DNA damage-binding protein 2; PARG, poly(ADP-ribose) glycohydrolase; PCNA, proliferating cell nuclear antigen; Pol δ/ε/κ, DNA polymerase δ, ε, and κ; SSB, single-strand break; TFIIH, transcription factor IIH; XPA, xeroderma pigmentosum group A-complementing protein; XPC, xeroderma pigmentosum group C-complementing protein; XRCC1, X-ray repair cross-complementing protein 1. Created in Biorender. Jang, S. (2026) https://BioRender.com/zve6buf.

**Figure 3:**
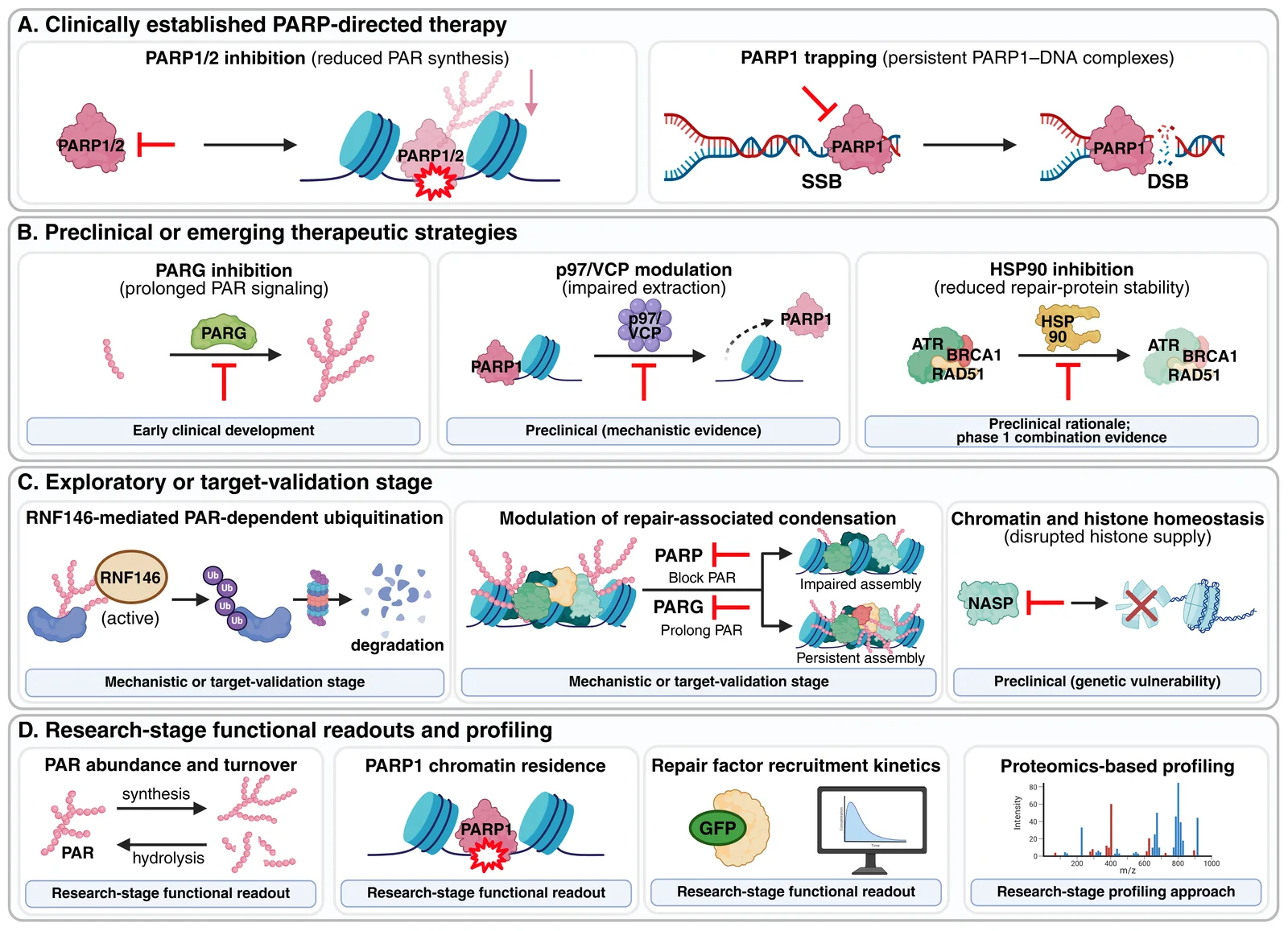
Therapeutic and research-stage implications of PAR-associated repair-protein dynamics in cancer. (**A**) Clinically established PARP-directed therapy reduces PAR synthesis and can promote persistent PARP1–DNA complexes through PARP1 trapping. (**B**) Emerging therapeutic strategies include PARG inhibition, which prolongs PAR signaling; p97/VCP modulation, which impairs extraction of persistent chromatin-bound PARP1; and HSP90 inhibition, which destabilizes repair and checkpoint proteins. (**C**) Exploratory or target-validation-stage concepts include RNF146-mediated PAR-dependent ubiquitination, modulation of repair-associated condensation through altered PAR metabolism, and NASP-dependent histone homeostasis. RNF146-dependent PAR recognition and NASP-dependent histone regulation are mechanistically established, whereas pharmacological control of repair-associated condensation remains less established. Their extension to BER/NER repair-protein turnover, condensation-directed therapy, or clinically tractable NASP targeting remains unproven. (**D**) PAR abundance and turnover, PARP1 chromatin residence, repair-factor recruitment kinetics, and proteomics-based profiling represent research-stage functional readouts or profiling approaches whose clinical utility remains to be established. Abbreviations: ATR, ataxia telangiectasia and Rad3-related; BRCA1, breast cancer gene 1; DSB, double-strand break; GFP, green fluorescent protein; HSP90, heat shock protein 90; NASP, nuclear autoantigenic sperm protein; PARG, poly(ADP-ribose) glycohydrolase; p97/VCP, p97/valosin-containing protein; RNF146, Ring finger protein 146; Ub, ubiquitin. Created in Biorender. Jang, S. (2026) https://BioRender.com/9l21qez.

**Table 1:** Selected mechanistic relationships between PAR signaling and repair protein dynamics in BER and NER.

Pathway	Protein	Relationship with PAR Signaling	Experimental Basis	Protein-Dynamic Consequence	Proteostatic Mechanism
BER/SSB repair	PARP1	Direct PAR-dependent through auto-ADP-ribosylation	• Site-specific auto-ADP-ribosylation studies • Biochemical/single-molecule and live cell measurements of DNA association • PARPi perturbation	DNA dissociation, chromatin residence, and PARPi-induced retention/trapping	SUMO–RNF4–UFD1–p97/VCP-dependent chromatin extraction
	XRCC1	Direct PAR-dependent through PAR binding by BRCT1	• PAR-binding mutants • PARP inhibition • Laser damage/live cell recruitment assays	Rapid recruitment and retention at SSB/BER intermediates	HSP90-dependent stabilization of the XRCC1–Pol β complex
BER	Pol β	PAR-associated, principally through XRCC1-centered BER organization	• XRCC1–Pol β interaction and BER-complex studies	Assembly within BER complexes and regulation of functional protein availability	Mule/ARF–BP1- and CHIP-mediated ubiquitin-dependent abundance control
	FEN1	Subject to broader proteostatic regulation	• Biochemical and cellular analyses of sequential PTMs	Chromatin association and protein turnover	Sequential phosphorylation, SUMOylation, ubiquitination, and proteasomal turnover
	MUTYH	Subject to broader proteostatic regulation	• Ubiquitination-deficient mutants • Protein stability and chromatin-association assays	Protein stability and chromatin engagement	Mule-dependent ubiquitination and stability control
GG-NER	DDB2	PARP1-associated; DDB2 ADP-ribosylation demonstrated	• PARP1–DDB2 interaction • DDB2 ADP-ribosylation • Chromatin retention and protein stability analyses	Lesion-site retention, stability, and transition to downstream NER factors	CRL4^DDB2-dependent ubiquitination, factor displacement, and proteasomal turnover
	ALC1	Direct PAR-dependent through its macrodomain	• PAR-binding assays • PARP inhibition • Live cell damage site recruitment • Biochemical nucleosome-remodeling assays	Rapid recruitment, transient residence, and chromatin remodeling	Chromatin recruitment and release coupled to PAR-dependent remodeling
	XPC	PAR-associated and PARP1-dependent	• PARP1–XPC interaction studies • Perturbation of PARP catalytic activity • Cellular damage site recruitment studies	Recruitment and stabilization at UV lesions; transition to damage verification	Ubiquitin-dependent regulation of lesion residence and factor exchange
	XPA	Subject to broader proteostatic regulation	• Phosphorylation mutants • HERC2 interaction studies • Ubiquitination and protein stability assays	Protein stability and availability for pre-incision complex formation	ATR/HERC2-regulated ubiquitination and protein stability
TC-NER	CSB	Direct PAR-dependent in the early TC-NER response	• PARP/PARG perturbation • PARylation-resistant CSB mutants • CSB–RNAPII interaction and repair assays	Stabilization at lesion-stalled RNAPII and modulation of CSB ATPase activity	Ubiquitin-dependent regulation of stalled transcription complexes

Notes: Direct PAR-dependent indicates protein-specific regulation experimentally linked to PAR binding, ADP-ribosylation, or selective perturbation of PAR synthesis or hydrolysis. PAR-associated indicates regulation occurring within a PAR-regulated repair environment without a defined direct molecular connection to PAR. Broader proteostatic regulation indicates chaperone-, ubiquitin-, extraction-, or degradation-dependent control without demonstrated PAR initiation. Abbreviations: CRL4^DDB2, DDB2-containing cullin 4-RING E3 ubiquitin ligase complex.

**Table 2:** Translational status of therapeutic strategies and functional readouts related to PAR-associated repair-protein dynamics.

Strategy or Readout	Mechanistic Basis	Translational Status	Relevant Setting	Principal Limitation
PARP inhibitors	• Blockade of PAR synthesis • PARP1 trapping	Clinically established	*BRCA1/2*-altered and HR-deficient cancers	• Acquired resistance • Context-dependent trapping
PARG inhibition	• Prolonged PAR signaling • Sensitization to replication stress	Early clinical development	HR-deficient and tumors associated with replication stress	• Uncertain predictive biomarkers • Incompletely defined therapeutic window
p97/VCP-dependent extraction	• Impaired removal of trapped PARP1 from chromatin	Preclinical mechanistic rationale	PARPi-treated, HR-defective models	• Pleiotropic essential functions • Limited selectivity
HSP90 inhibition	• Destabilization of HR and checkpoint proteins	Preclinical rationale with phase 1 combination evaluation	HR-restored or chaperone-dependent tumors	• Broad client repertoire • Limited repair specificity
Histone homeostasis /NASP	• Histone buffering • Chromatin adaptation	Preclinical genetic vulnerability	PARPi-resistant models	• Limited pharmacological tractability • Lack of clinical validation
Repair-associated condensation	• Altered local protein concentration, partitioning, and exchange	Exploratory mechanistic axis	Reconstituted and selected cellular systems	• Uncertain causal role in repair • Lack of selective modulators
PAR abundance /PARP1 chromatin association	• Functional measurement of PAR metabolism and PARP1 residence	Research-stage functional readout	Translational studies of PARP/PARG inhibitors	• Rapid PAR turnover • Sample-processing variability • Lack of assay standardization
Repair-factor recruitment and residence kinetics	• Dynamic measurement of XRCC1, DDB2, and related repair factors	Research-stage functional readout	Live-cell, ex vivo, and organoid models	• Requirement for viable specimens • Need for controlled DNA damage induction • Lack of standardized imaging protocols

## Data Availability

No new data were created or analyzed in this study. Data sharing is not applicable to this article.

## Abbreviations

3BP2	SH3 domain-binding protein 2
ALC1	amplified in liver cancer 1
APE1	apurinic/apyrimidinic endonuclease 1
ARF-BP1	alternative reading frame-binding protein 1
ARH3	ADP-ribosylhydrolase 3
ATR	ataxia telangiectasia and Rad3-related
AXIN	axis inhibition protein
BER	base excision repair
BP1	binding protein 1
BRCA1	breast cancer gene 1
BRCA2	breast cancer gene 2
BRCT1	first BRCA1 C-terminal domain
CHIP	C-terminus of Hsc70-interacting protein
CHK1	checkpoint kinase 1
CRL4^DDB2	DDB2-containing cullin 4-RING E3 ubiquitin ligase complex
CSA	Cockayne syndrome group A protein
CSB	Cockayne syndrome group B protein
DDB1	DNA damage-binding protein 1
DDB2	DNA damage-binding protein 2
DDR	DNA damage response
DNA-PKcs	DNA-dependent protein kinase catalytic subunit
DSB	double-strand break
FEN1	flap endonuclease 1
FRAP	fluorescence recovery after photobleaching
FUS	fused in sarcoma
GG-NER	global genome nucleotide excision repair
HERC2	HECT and RLD domain-containing E3 ubiquitin protein
hnRNP A1	heterogeneous nuclear ribonucleoprotein A1
HPF1	histone PARylation factor 1
HR	homologous recombination
HRD	homologous recombination deficiency
HSP90	heat shock protein 90
MUTYH	mutY DNA glycosylase homolog
NAD^+^	nicotinamide adenine dinucleotide
NASP	nuclear autoantigenic sperm protein
NER	nucleotide excision repair
NHEJ	non-homologous end joining
OGG1	8-oxoguanine DNA glycosylase 1
PAR	poly(ADP-ribose)
PARdU	poly(ADP-ribose)-dependent ubiquitination
PARG	poly(ADP-ribose) glycohydrolase
PARP	poly(ADP-ribose) polymerase
PARP1	poly(ADP-ribose) polymerase 1
PARP2	poly(ADP-ribose) polymerase 2
PARPi	PARP inhibitor
PARylation	poly(ADP-ribosyl)ation
PNKP	polynucleotide kinase 3′-phoaphatase
Pol β	DNA polymerase beta
Pol δ/ε/κ	DNA polymerase delta/epsilon/kappa
PTMs	post-translational modifications
p97/VCP	p97/valosin-containing protein
RNAPII	RNA polymerase II
RNF4	Ring finger protein 4
RNF146	Ring finger protein 146
SSB	single-strand break
SUMO	small ubiquitin-like modifier
TC-NER	transcription-coupled nucleotide excision repair
TDP-43	TAR DNA-binding protein 43
TFIIH	transcription factor IIH
UFD1	ubiquitin fusion degradation protein 1
UV	ultraviolet
UV-DDB	ultraviolet-damaged DNA-binding protein complex
WWE	tryptophan–tryptophan–glutamate
XPA	xeroderma pigmentosum group A-complementing protein
XPC	xeroderma pigmentosum group C-complementing protein
XRCC1	X-ray repair cross-complementing protein 1
